# Pulmonary metastasis with coexisting pulmonary mucosa‐associated lymphoid tissue (MALT) lymphoma 20 years after endometrioid adenocarcinoma surgery: A case report

**DOI:** 10.1111/1759-7714.13776

**Published:** 2020-12-09

**Authors:** Daisuke Nakamura, Nobutaka Kobayashi, Masahisa Miyazawa, Kentaro Miura, Hidetoshi Satomi

**Affiliations:** ^1^ Department of Thoracic Surgery Japanese Red Cross Society Nagano Hospital Nagano Japan; ^2^ Division of General Thoracic Surgery, Department of Surgery Shinshu University School of Medicine Matsumoto Japan; ^3^ Department of Pathology Japanese Red Cross Society Nagano Hospital Nagano Japan

**Keywords:** Endometrioid adenocarcinoma, late recurrence, lung metastases, mucosa‐associated lymphoid tissue (MALT) lymphoma

## Abstract

Late pulmonary metastasis from endometrioid adenocarcinoma (EA) is rare, and occurrence after >20 years is extremely rare. Here, we report a case of pulmonary metastasis with coexisting pulmonary mucosa‐associated lymphoid tissue (MALT) lymphoma that occurred 20 years after surgery for EA. A 60‐year‐old Japanese woman had previously undergone surgery for primary EA, and 20 years later presented with an abnormality that was detected on chest radiography. Chest computed tomography (CT) revealed two nodules in the right lower lung lobe, which were suspected to be primary lung cancer. Wedge resection was performed, and the intraoperative pathological diagnosis was that of adenocarcinoma with MALT lymphoma; this prompted additional right lower lobectomy. The final pathological diagnosis was pulmonary metastasis from EA with coexisting MALT lymphoma. This is probably the first report on late pulmonary metastasis coexisting with MALT lymphoma 20 years after surgery for EA. Surgeons should be aware of the possibility of late pulmonary recurrence of EA after more than 20 years and should consider aggressive resection.

**Key points:**

**Significant findings of the study:**

Although extremely rare, pulmonary metastasis can occur more than 20 years after surgery for endometrioid adenocarcinoma. Furthermore, pulmonary metastasis from endometrioid adenocarcinoma may coexist with mucosa‐associated lymphoid tissue lymphoma.

**What this study adds:**

Endometrioid adenocarcinoma requires long‐term postoperative follow‐up to detect recurrence, even in early‐stage cases. Video‐assisted thoracoscopic surgery (VATS) is useful for resecting pulmonary metastasis from endometrioid adenocarcinoma.

## Graphical abstract

Pulmonary recurrence from endometrioid adenocarcinoma (EA) after more than 20 years is extremely rare. We report a case of pulmonary metastasis with coexisting pulmonary mucosa‐associated lymphoid tissue (MALT) lymphoma 20 years after surgery for primary EA. Late pulmonary recurrence of EA (even after >20 years) should be considered during treatment planning for even early‐stage EA, and surgeons should consider aggressive resection based on this possibility.

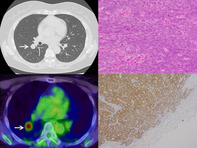

## Introduction

Pulmonary metastasis is rarely reported more than 20 years after treatment for endometrioid adenocarcinoma (EA).^1^ Primary pulmonary mucosa‐associated lymphoid tissue (MALT) lymphoma, a low grade B‐cell lymphoma, is also rare, and accounts for less than 0.5% of all lung tumors.^2^ To the best of our knowledge, there have been no reports on pulmonary metastasis coexisting with MALT lymphoma more than 20 years after surgical treatment for EA.

## Case report

**Figure 1:**
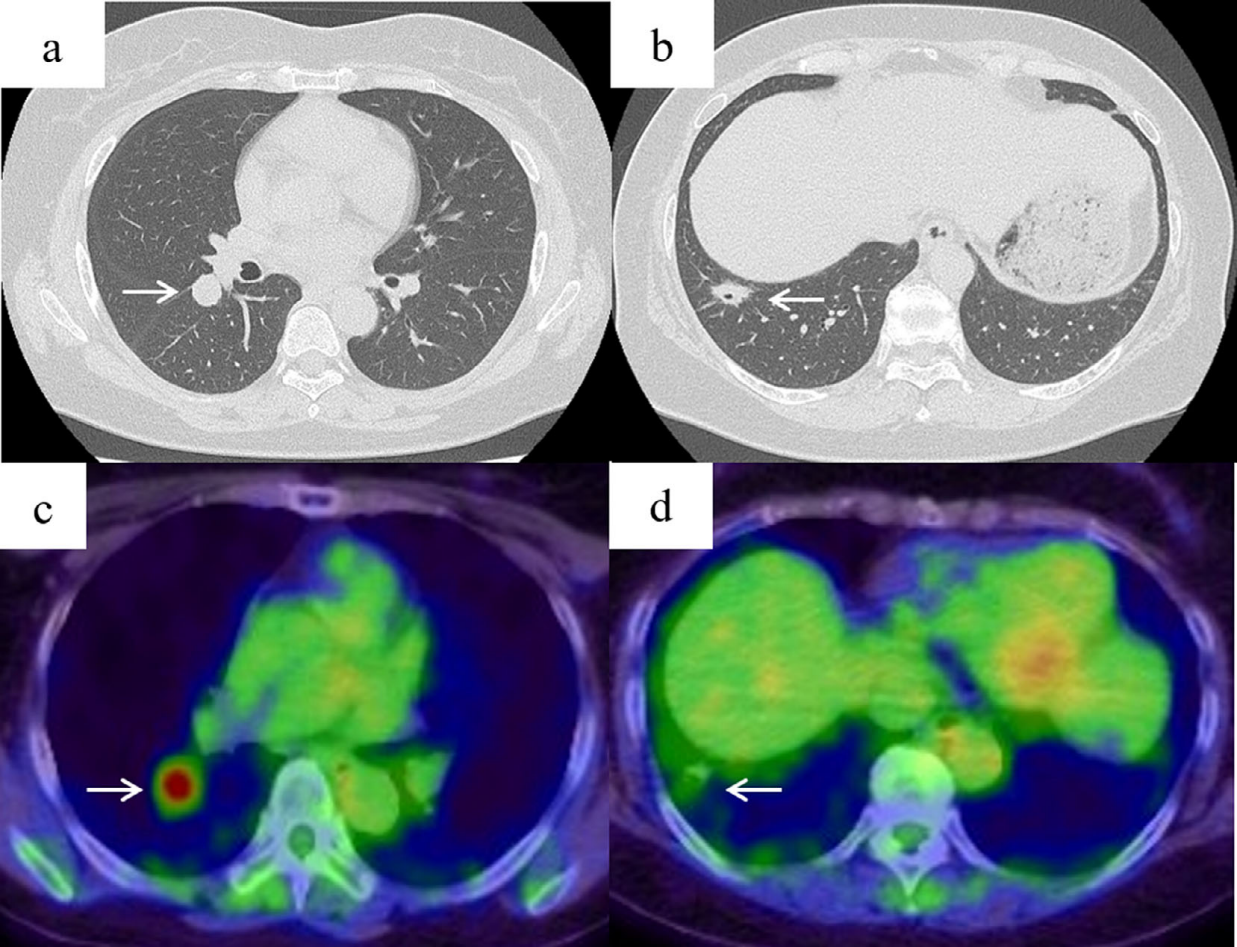
Chest computed tomography (CT) showed two nodules in the right lower lobe (arrow). (**a**) A well defined nodule; and (**b**) an irregularly‐defined nodule on air bronchograms. Both nodules exhibited tracer uptake (arrow) during 2‐[^18^F]‐fluoro‐2‐deoxyglucose positron emission tomography‐computed tomography, with (**c**) a maximum standardized uptake value (SUVmax) of 6.6; and (**d**) a SUVmax value of 2.0.

A 60‐year‐old non‐smoking Japanese woman was referred to our hospital because of an abnormality detected on chest radiography. Twenty years earlier, she had undergone total abdominal hysterectomy and bilateral salpingo‐oophorectomy with pelvic lymphadenectomy for EA (FIGO stage: IA, histological grade: G1). She had not received any adjuvant therapy for the EA, and no signs of recurrence were detected during follow‐up at five years after surgery. At the current admission, the findings from physical examination and laboratory testing were considered normal. Chest computed tomography (CT) revealed a well‐defined pulmonary nodule (diameter: 17 mm) and an irregularly‐defined nodule (diameter: 22 mm), with air bronchograms in the right lower lobe (Fig 1a,b). The nodules exhibited tracer uptake during 2‐[^18^F]‐fluoro‐2‐deoxyglucose positron emission tomography (FDG‐PET)‐CT, with maximum standardized uptake values (SUVmax) of 6.6 and 2.0 in the well‐ and irregularly‐defined nodules, respectively (Fig 1c,d). FDG‐PET‐CT findings did not reveal any accumulation indicative of local recurrence of EA. We suspected double primary lung cancers and performed wedge resection for the two nodules using video‐assisted thoracoscopic surgery (VATS). The intraoperative pathological diagnosis of the well‐defined nodule was adenocarcinoma and that of the irregularly‐defined nodule was MALT lymphoma. Thus, we also performed right lower lobectomy with hilar and mediastinal lymph dissection.

**Figure 2:**
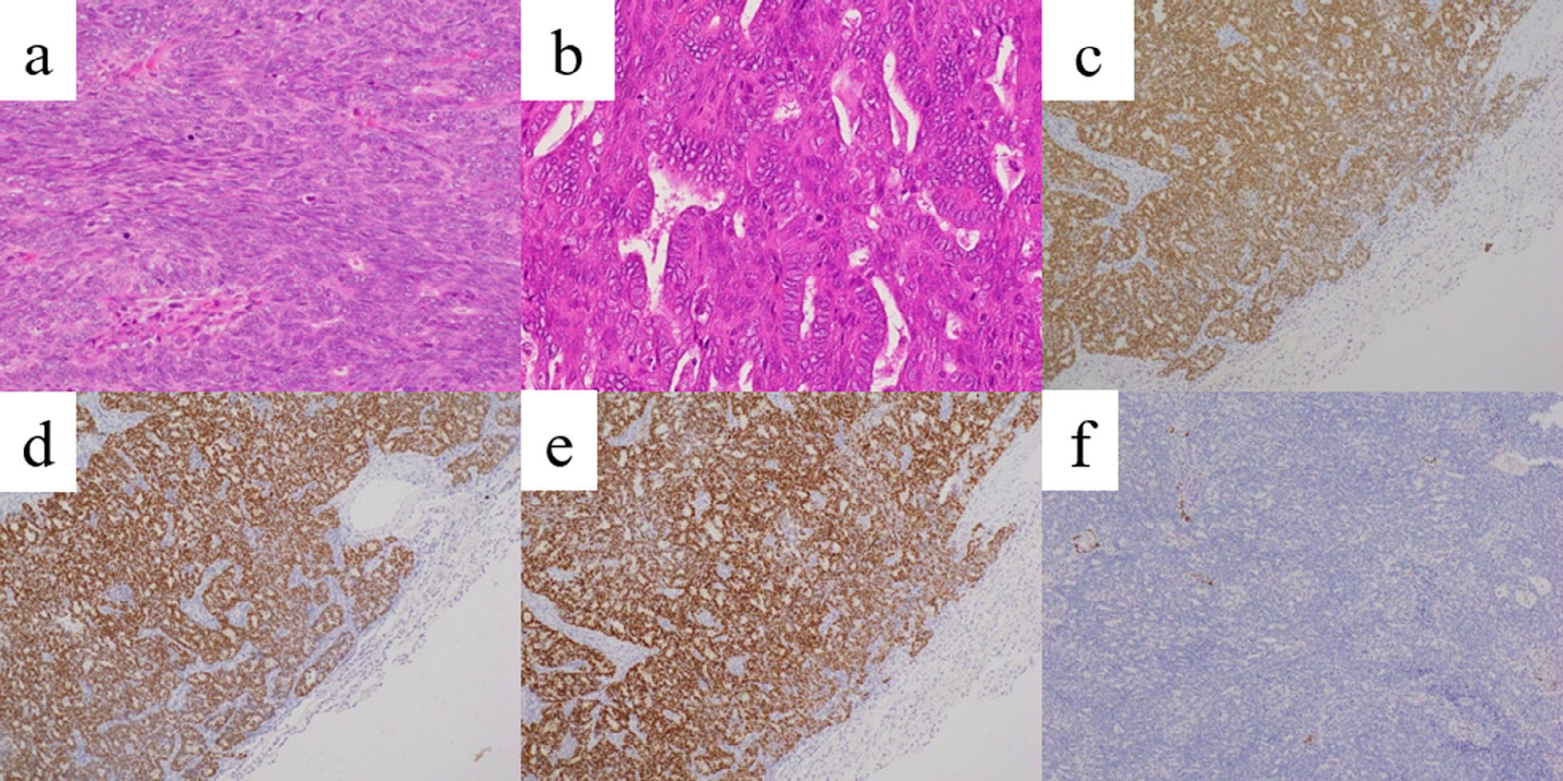
Histological examination of the resected well defined nodule showing tumor cells with crude chromatin proliferation in various forms. The pathological diagnosis was that of adenocarcinoma and the specimen was very similar to the endometrial adenocarcinoma tissue that had been resected 20 years earlier (hematoxylin and eosin staining; **a**: lung tissue, ×100, **b**: uterine tissue, ×400). The tumor cells were immunohistochemically positive for (**c**) paired‐box gene 8 (PAX8), (**d**) estrogen receptor (ER), and (**e**) progesterone receptor (PR), but negative for (**f**) thyroid transcription factor‐1 (TTF‐1) (×40).

**Figure 3:**
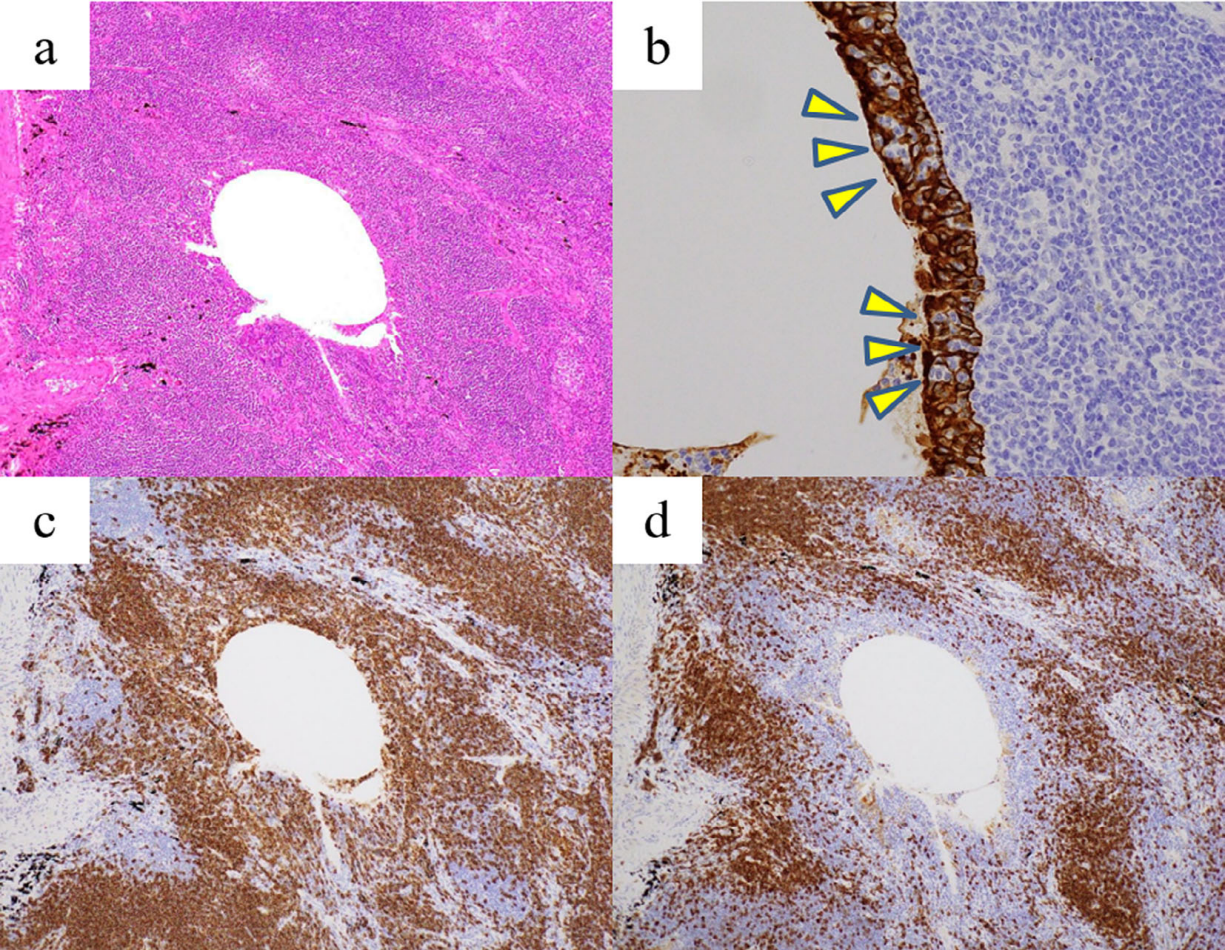
Histological examination of the resected irregularly‐defined nodule showing small to medium‐sized infiltrating lymphocytes (**a**: hematoxylin and eosin staining, ×100) and lymphoepithelial lesions in the bronchial epithelium (**b**: arrowhead, AE1/AE3 staining, ×400). These lymphocytes were immunohistochemically positive for (**c**) CD20; and negative for (**d**) CD5 (×100).

Histological examination of tissue from the resected well‐defined nodule revealed adenocarcinoma; this was very similar to the EA tissue that had been resected 20 years earlier (Fig 2a,b). Immunohistochemistry revealed that the tumor cells were positive for paired‐box gene 8 (PAX8), estrogen receptor (ER), and progesterone receptor (PR), but negative for thyroid transcription factor‐1 (TTF‐1) (Fig 2c–f). Histological examination of tissue from the resected irregularly‐defined nodule revealed small to medium‐sized infiltrating lymphocytes and lymphoepithelial lesions in the bronchial epithelium (Fig 3a,b). Immunohistochemistry revealed that these lymphocytes were positive for CD20 and negative for CD5 (Fig 3c,d). Therefore, the final pathological diagnosis was that of pulmonary metastasis from EA with coexisting MALT lymphoma. The postoperative course was uneventful and the patient exhibited no signs of recurrence at one‐year postoperative follow‐up without any adjuvant therapy.

## Discussion

The incidence of pulmonary metastasis from endometrial cancer including EA has been reported to be approximately 2.3%–7%.^3^ However, recurrence of EA usually occurs within three years after initial treatment.^4^, ^5^ Among malignant tumors of the uterus, leiomyosarcoma and endometrial stromal sarcoma have been reported sporadically at more than 20 years after surgery,^6^, ^7^ but late pulmonary metastasis from EA at more than 20 years after surgery is extremely rare.^1^ Miyamoto *et al*. reported a case of lung metastasis at 10 years after endometrial cancer surgery.^8^ However, this was a relatively advanced cancer (FIGO stage: IIIC, histological grade G3); our case had early stage EA (FIGO stage: IA, histological grade G1). Thus, we did not suspect metastatic pulmonary tumors in the preoperative differential diagnosis and performed pulmonary resection to diagnose and treat suspected primary lung cancer. The well‐defined nodule was eventually diagnosed as pulmonary metastasis from EA; this was supported by the positive expression of PAX8 (expressed in 98% of EAs).^9^ Good prognostic factors for patients with pulmonary metastasis from uterine malignancies include (i) a grade 1–2 primary tumor; (ii) a maximum pulmonary tumor diameter of <2 cm' (iii) positive ER status; (iv) a disease‐free interval of >12 months; and (v) <3 metastases.^3^, ^10^ Our patient had all of these factors and her prognosis was considered good. Surgical resection is a viable option in cases of pulmonary metastasis, including from uterine cancers, and pulmonary metastasectomy using VATS is a safe and feasible procedure in cases with primary uterine malginancies.^11^

Primary pulmonary MALT lymphoma is a rare low‐grade B‐cell lymphoma that accounts for <0.5% of all lung tumors.^2^ The prognosis of pulmonary MALT lymphoma is good, with a previously reported five‐year survival rate of 84%.^12^ However, pulmonary MALT lymphoma presents with diverse findings on CT,^13^, ^14^ and it can be difficult to achieve a preoperative diagnosis; it is often diagnosed after surgery. In our case, CT revealed an irregularly‐defined nodule with air bronchograms, which led us to suspect primary lung cancer. Nevertheless, the pathological findings were indicative of MALT lymphoma in the irregularly‐defined nodule; this surprisingly coexisted with pulmonary metastasis from EA. There are reports of pulmonary MALT lymphoma coexisting with primary lung cancer or infectious diseases;^15^, ^16^ however, reports on coexistent pulmonary MALT lymphoma and metastatic lung tumors are lacking.

In conclusion, to the best of our knowledge, this is the first report on late pulmonary metastasis coexisting with MALT lymphoma 20 years after surgery for EA. Surgeons should be aware of the possibility of late pulmonary metastasis from EA, even at more than 20 years after the original surgery, and should consider aggressive resection.

## Disclosure

The authors do not have any conflicts of interest to declare.
